# Community hospitals of the future: the role of community hospitals to mitigate health system burden in Singapore

**DOI:** 10.3389/frhs.2024.1407528

**Published:** 2024-07-23

**Authors:** Chuan De Foo, Hui Xiang Chia, Sherianne Yen Tze Tan, Yi Feng Lai, Jia En Joy Khoo, Shi Yun Tee, Cher Wee Lim, Ken Wah Teo

**Affiliations:** ^1^ Saw Swee Hock School of Public Health, National University of Singapore, Singapore, Singapore; ^2^ Duke NUS Graduate Medical School, Singapore, Singapore; ^3^ Lee Kuan Yew School of Public Policy, National University of Singapore, Singapore, Singapore; ^4^ MOH Office for Healthcare Transformation, Singapore, Singapore; ^5^ School of Public Health, University of Illinois, IL, United States; ^6^ Department of Pharmacy, Faculty of Science, National University of Singapore, Singapore, Singapore; ^7^ Division of Nursing, Changi General Hospital, Singapore, Singapore; ^8^ Preventive Medicine, National University Health System, Singapore, Singapore; ^9^ Division of Population Health and Integrated Care, Singapore General Hospital, Singapore, Singapore

**Keywords:** community hospital, right-site, chronic disease, health policy, health system reform

## Abstract

In Singapore, an ageing population with increasing chronic disease burden and complex social circumstances have strained the healthcare system. For the health system to run more efficiently, patients should be appropriately sited according to their medical needs. In Singapore, community hospitals serve as an intermediate inpatient facility managing patients with sub-acute and rehabilitation care needs. Our policy brief uncovers the gaps in transforming community hospital care models and offers actionable steps to unlock the community hospital chokepoints in Singapore's health system. The future community hospitals can accommodate higher acuity but medically stable patients, while patients who do not require inpatient rehabilitation care can be appropriately sited to community partners, if policy, resourcing and technology factors are addressed. An evidence-based, stepwise approach involving all stakeholders will be required to pilot and evaluate new models before large-scale change.

## 1 Introduction

Singapore is fast becoming a super-aged society, with at least 21% of its population over the age of 65 (1). The rising prevalence of chronic diseases and increasing social needs have, in turn, contributed to more complex patient profiles (2). This has led to increased acute hospital (AH) admissions from 105 to 130 per 1,000 population between 2013 and 2023 (3) and the average hospitalization episode lengthening from 6.1 days to 7 days between 2020 and 2023 (4), with more intensive service provision, a prolonged clinical recovery phase, and greater needs for functional optimization and care coordination. Healthcare costs are projected to rise from SGD $3.7 billion to $27 billion between 2009 and 2030 (5). Singapore needs to develop innovative models of care that better serve elderly patients and ensure healthcare costs remain sustainable (6).

Policy dialogues are emerging as a key tool for translating knowledge into policymaking. A policy dialogue is a mechanism for stakeholders to interact and share knowledge. Through these discussions, knowledge from research and the local environment is combined to make evidence-informed, context-specific policymaking possible (7). Workshops on systems thinking coupled with proactive policy engagement can also aid researchers and policymakers in unravelling the dynamic phenomenons observed in the healthcare landscape (8). Therefore, such policy dialogue and workshops when run in parallel, are useful for major public health problems, as the issues tackled often have conflicting stakeholder interests and no clear answer.

In 2023, Singapore's Ministry of Health (MOH) and MOH Office for Healthcare Transformation organised a series of policy dialogues and workshops, which brought together policymakers and stakeholders from all Regional Health Systems (RHSs), AHs, and community hospitals (CHs) to discuss how CHs can play a larger role in optimizing the health system in the face of rising demand from an ageing population. A 4-day design-, systems- and complexity-thinking workshop was conducted to envision the CH of the future through a structured process of identifying and prioritizing pain points, designing new models of care, and defining the policy shifts required to implement these solutions. Ninety-seven stakeholders, including medical, nursing, allied health, operations and senior management, participated in the dialogues and workshop. In this policy brief, we discuss the lessons which have emerged from this process and the potential of CHs to play a larger role by taking on some of the AH load, thereby alleviating health system strain.

## 2 Singapore's health system at a bird's eye view

Singapore's health system is divided into three RHSs based on geographical boundaries. Each RHS is managed by a Cluster, which is responsible for the health and well-being of the entire population within their region (9). The Cluster comprises AHs, CHs, polyclinics which are government-owned primary care clinics, specialist outpatient clinics (SOCs), and ambulatory surgical centers. The Cluster works with all health and social care providers, including those from the private and social service sectors such as General Practices (GPs), nursing homes (NHs), non-profit organizations, and social service agencies (10). Clusters are funded on a per-capita basis for all residents within their region and have autonomy to allocate resources to the sites of care within the region.

AHs are government-funded public hospitals which provide acute inpatient specialist care. AHs are under pressure, with bed occupancy consistently above 90% and median bed waiting time 40% higher than pre-pandemic levels (11). Bed demand is expected to increase with the ageing population. Furthermore, there are supply-side constraints, including healthcare manpower shortages and long lag times to build new capacity in AH and downstream including community service providers and NHs.

CHs function as a transitional inpatient facility for patients requiring time-limited convalescent care after they have been stabilized in an AH (12). In line with this, CHs provide rehabilitative and sub-acute care services to enable patients to return home and resume their daily activities promptly (13). Consequently, CHs are expected to assume a progressively crucial role in delivering care for an ageing population.

However, since the first CH was built 30 years ago, national regulation on the scope of CH services has not been updated sufficiently to cater to the changing demographics. Several local studies have shown that 30–40% of general medicine and geriatric patients in AHs today have subacute rather than acute needs and could be right-sited to CHs. Despite this, AH to CH transfer rates are only 4%–5%, with a lengthy 1-week wait time from referral to transfer (14, 15). This problem is exacerbated by patient inflows to CH being directed through the AH Emergency Department (ED) and inpatient wards, rather than direct admission to CH, even if the patient only requires CH care. While a direct admission option from the ED is available, only about three patients are referred each month nationally. Additionally, the average CH length of stay is substantially longer than that of AH (31 days vs. 5 days) despite high compliance to care processes such as early review by a senior doctor, multidisciplinary care, and early discharge planning. Downstream, there are non-medical reasons for delays in discharging patients, including insufficient NH capacity, and difficulty arranging for a caregiver at home. This results in a chokepoint in the health system. These patient flows are summarized in Supplementary Figure S1 in the appendix.

## 3 Policy recommendations and implications

Several policy options to improve health system throughput by leveraging CH capabilities and capacity were explored during the dialogues and workshop. Below, we discuss seven recommendations across the AH—CH—home care continuum. Each recommendation addresses key pain points and builds on the existing good practices across institutions, as summarized in Supplementary Figure S2 of the appendix.

### 3.1 CH doubling as a potential gatekeeper to higher care levels

Patients who could be managed in CH could be diverted away from AH and instead directly sited in CH, lessening the load placed on AH and, in turn, reducing bed occupancy rates and waiting times for admissions. The primary and community care landscape is undergoing a major transformation called Healthier SG (16). Family Physicians in polyclinics, GPs in private practices and community health and social care providers have been tasked with greater responsibility to foster longitudinal relationships with residents and be upskilled with standardized care protocols integrated across the health system. These reforms provide an opportunity to insert direct admission pathways to CHs within the network of primary and community health services, enabling gatekeeping to prevent unnecessary admissions to AHs for patients who could be appropriately cared for in CHs. Clear triaging, referral, care, escalation and transition protocols should be in place to ensure patient outcomes and safety and prevent over- or under-servicing. Efforts must also raise awareness among primary care providers on the availability of this option. This policy shift would also require timely feedback mechanisms and strong trust and communication between all parties in the ecosystem. Finally, subsidies would need to be extended to these new admission routes to remove an otherwise prohibitive financial barrier for transition to step-down care programs from the patient's perspective (17, 18).

In the long run, CHs could serve as the first “step-up” from the community where patients would, as a default, be admitted to CH rather than AH, unless otherwise contra-indicated. To enable this shift, the use of strict inclusion criteria for CH admissions should shift to an exclusion-based criteria, which allows for a broader range of patients to be admitted.

### 3.2 Manage higher acuity of patients in CH

In tandem with expanding direct admission sources to CH, more frequent and earlier transfers from AHs can be encouraged by allowing and enabling CHs to manage a higher acuity of patients. The CH Clinical Services Manual, which regulates the allowable CH patient mix and service scope, excludes all patients with acute care needs. These include patients who: (i) may not have a definitive diagnosis and require additional advanced investigations, (ii) have pending advanced investigations planned, and (iii) require unplanned advanced investigations to avoid AH readmission for conditions. These patients would necessarily be clinically stable and at low risk of deterioration, and CHs determined to be within their technical capability as a Family Physician- and Geriatrician-led service to manage. To achieve this, AHs and CHs would need to foster tighter partnerships through shared care and the development of common and integrated care protocols. There is also a need to communicate the CH scope of services and capabilities to AHs to prevent inappropriate referrals, such as for patients with only social issues, who should be right-sited directly into community-based social services. To manage this group of more complex patients who would not generally need inpatient specialist acute care, CHs would also need to be resourced with higher staff-to-bed ratios to cater to the need for increased intensity of clinical monitoring and management. At the policy level, CHs would need seamless access to subsidized advanced investigations sited at AHs and an updated CH service scope.

### 3.3 Optimise CH care model efficiency

#### 3.3.1 Upskilling healthcare staff

Clinical, pharmacy, and therapy services are limited on weekends due to existing CH funding norms and the exigencies that they face on a busy clinical workday. This limits AH—CH transfers, efficiency of CH care delivery and discharge planning. Staff-to-bed ratios would need to be reviewed, and funding increased in tandem with change management to provide this additional coverage. Additionally, a cost-effectiveness study of providing higher intensity, more optimized therapy and clinical monitoring in tandem with increasing staff-to-bed ratios could be considered. New AH—CH shared care pathways and initiating CH-level rehabilitation in AH while awaiting AH—CH transfer are other care redesign ideas that leverage collaboration between AH and CH teams. However, policymakers must also acknowledge the need to equitably distribute trained manpower resources to all areas of the health system and simultaneously roll out measures to retain existing healthcare workers (19).

#### 3.3.2 Harnessing technology

The COVID-19 pandemic has accelerated and normalised the use of technology to deliver remote care and services (20). Additionally, the next generation of young seniors is more health and digitally literate and can be empowered for self-care and ownership over their health. CHs should ride this wave and expand beyond their current physical boundaries, into homes and communities, and digital and virtual spaces. Telehealth services, including clinical consultation, monitoring and rehabilitation, and mobile services such as home or de-centralized intravenous antibiotic administration, wound care and complex therapy, can be provided by CHs. This could free up CH bed capacity by substituting a physical CH stay with an offsite one and supporting earlier discharge from CH. Rehabilitation technologies should also be incorporated into existing CH services at the CH, home or community, provided they are evidence-based and cost-effective, to improve care efficiency and facilitate early CH discharge (21).

### 3.4 Right-siting low intensity, uncomplicated rehabilitation patients to community partners

Improving CH throughput also requires expediting the discharge of CH patients who may be better suited for care in community and home-based settings. This can include patients who are non-weight bearing or require slow-stream, low-intensity rehabilitation. Importantly, proper post-discharge care provided at a patient's home has been shown to offer good patient outcomes (22). Clinical supervision can be provided on a consultation basis by the CH if required, but direct care should be delivered by community service providers. The success of such a model relies on timely, holistic and complete discharge planning in the AH, and a mutual understanding that patients should not be discharged to CH solely for care coordination and discharge planning. Additionally, tighter partnerships with mutually agreed arrangements between AH, CH and community partners should be fostered (23). Additionally, financial accessibility is a key barrier to patients receiving outpatient care. Subsidies and insurance coverage must incentivize appropriate siting of care.

### 3.5 Work with community partners, patients and caregivers to support early CH discharge

The ageing population has increasingly complex medical and social care needs, which require early and comprehensive care coordination and discharge planning. A collaborative approach between CH, community service providers, patients and their caregivers should be adopted, for example, through multi- and trans-disciplinary discussions centred around patient needs. For productive value co-creation to take place, the co-engineering process must be removed from power asymmetries and vested interests (24). Stakeholders should co-design care pathways which enable seamless transitions of care, facilitated by a shared understanding of each provider's capabilities and responsibilities and organized information flows about the patient's condition and care plans that are not inimical to any party. Loss to follow-up with community providers can result in patients deteriorating and U-turning back to AHs, and such partnerships must include standardized clinical pathways, protocols and feedback.

### 3.6 Evidence-based, stepwise approach to transformation

During the policy dialogues, participants identified several policy constraints which would hinder the implementation of new care models, including (i) the scope of allowable CH clinical services and associated infrastructure and equipment available to CHs, (ii) healthcare staffing, (iii) quantum and model of CH funding, and (iv) criteria for patient access to the national health insurance scheme, personal pension medical savings accounts, and government healthcare subsidies. These policy constraints would need to be reviewed in a safe space for experimentation, to pilot proposed changes and assess the evidence before enacting large-scale policy change. Upon completion of the policy dialogues, several proposals have been jointly selected by Cluster Senior Management, MOH and MOHT to undergo a MOH-endorsed sandbox to implement proof-of-concept pilots with programmatic funding and monitoring and evaluation support. A second proof-of-value phase would be considered, where successful elements of the care models tested are merged into a common care model to be tested at new sites, on new patient types, and at a larger patient volume, upon conclusion of the initial pilots. Details of the flow of events are summarized in Supplementary Figure S3 in the appendix.

### 3.7 Foster an ecosystem of government, cluster, AH, CH and community partners for the future

The success of CH reforms depends on the ability of all ecosystem stakeholders—government, Cluster, AH, CH and community partners, to align on a shared vision of optimizing the whole health system flow. The policy dialogues were a first step which brought together stakeholders to collectively identify important pain points and ideate future CH care models. These conversations must continue through formal platforms and informal avenues. To this end, the MOH-commissioned CH sandbox is supported by a tiered clinical and corporate governance framework to demonstrate clearer decision-making, accountability, and strategic alignment across all participating entities.

## 4 Conclusion

Our policy dialogue uncovered several care model-focused redesign options to enhance health system throughput by leveraging enhanced inflows to CH, augmenting CH capacity, improving CH care efficiency, and supporting a faster flow of patients down to the community. Underpinning these flows are several common approaches which necessitate closer relationships, clarified ways of working and timely feedback between all providers in the ecosystem, harnessing telemedicine and technology, and ensuring that policy appropriately enables and incentivizes appropriate, safe, quality and affordable care. The health system itself comprises many actors across different levels of system, and there is much heterogeneity within the CH space itself. This policy brief serves to collate a coherent set of priorities from the collective voices of diverse and powerful stakeholders, into an implementable, stepwise and evidence-based approach to transformation of CH care.
