# Supplementary material for: Community hospitals of the future: the role of community hospitals to mitigate health system burden in Singapore
Source: Front Health Serv. 2024 Jul 23;4:1407528. doi: 10.3389/frhs.2024.1407528 (PMC11300341; doi:10.3389/frhs.2024.1407528)
Supplement: Supplementary file 1 [file Datasheet1.docx]

Appendix - Supplementary Material

# Supplementary Figures

#
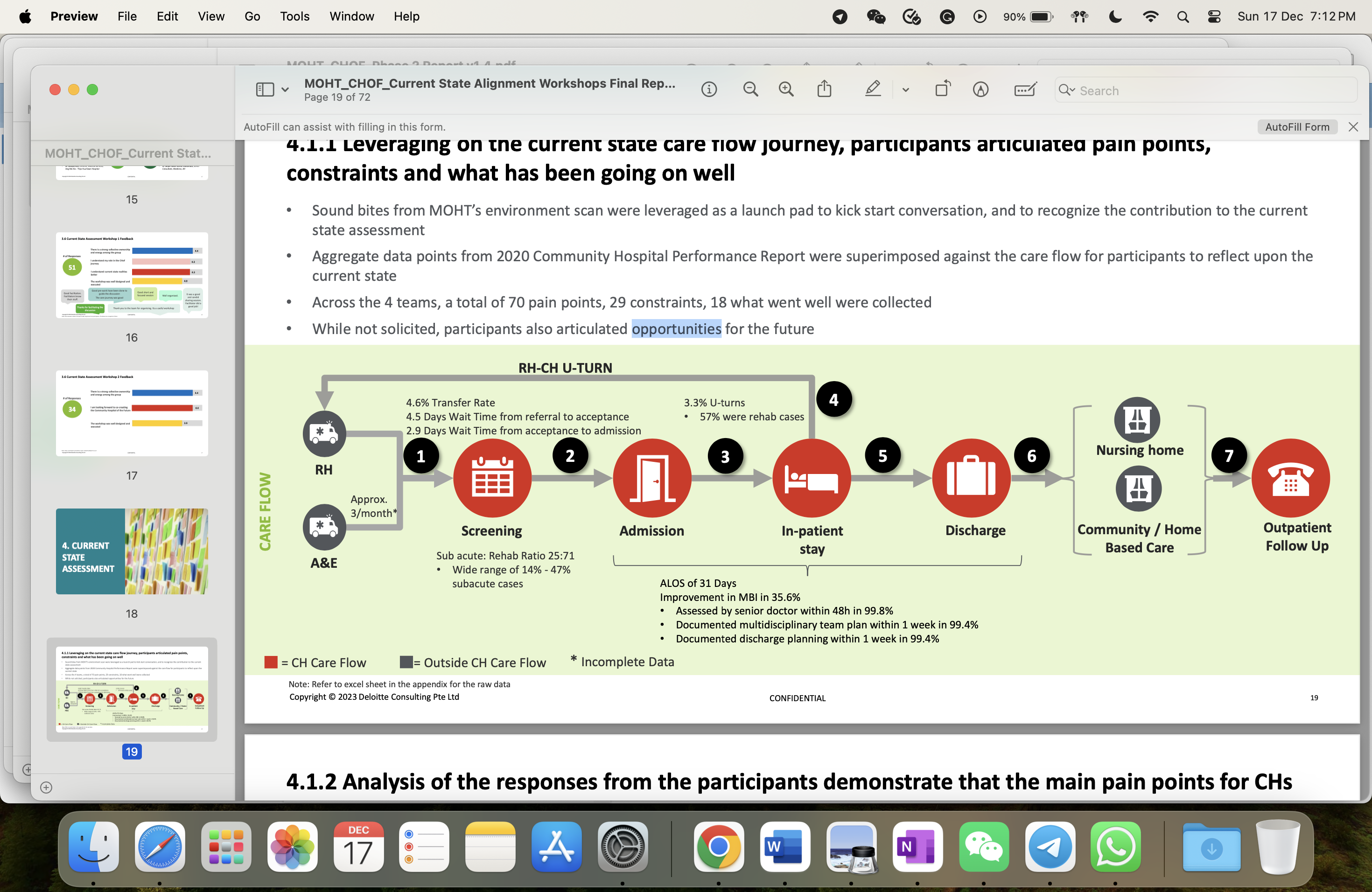


| **Legend:** | | | | | | |
| --- | --- | --- | --- | --- | --- | --- |
| **1** | **2** | **3** | **4** | **5** | **6** | **7** |
| Referral | Admission | In-patient stay | U-turn to acute hospital | Discharge | Nursing home, community/ home-based care | Outpatient follow-up |

**Figure 1.** Patient flow pathways for community hospital transition


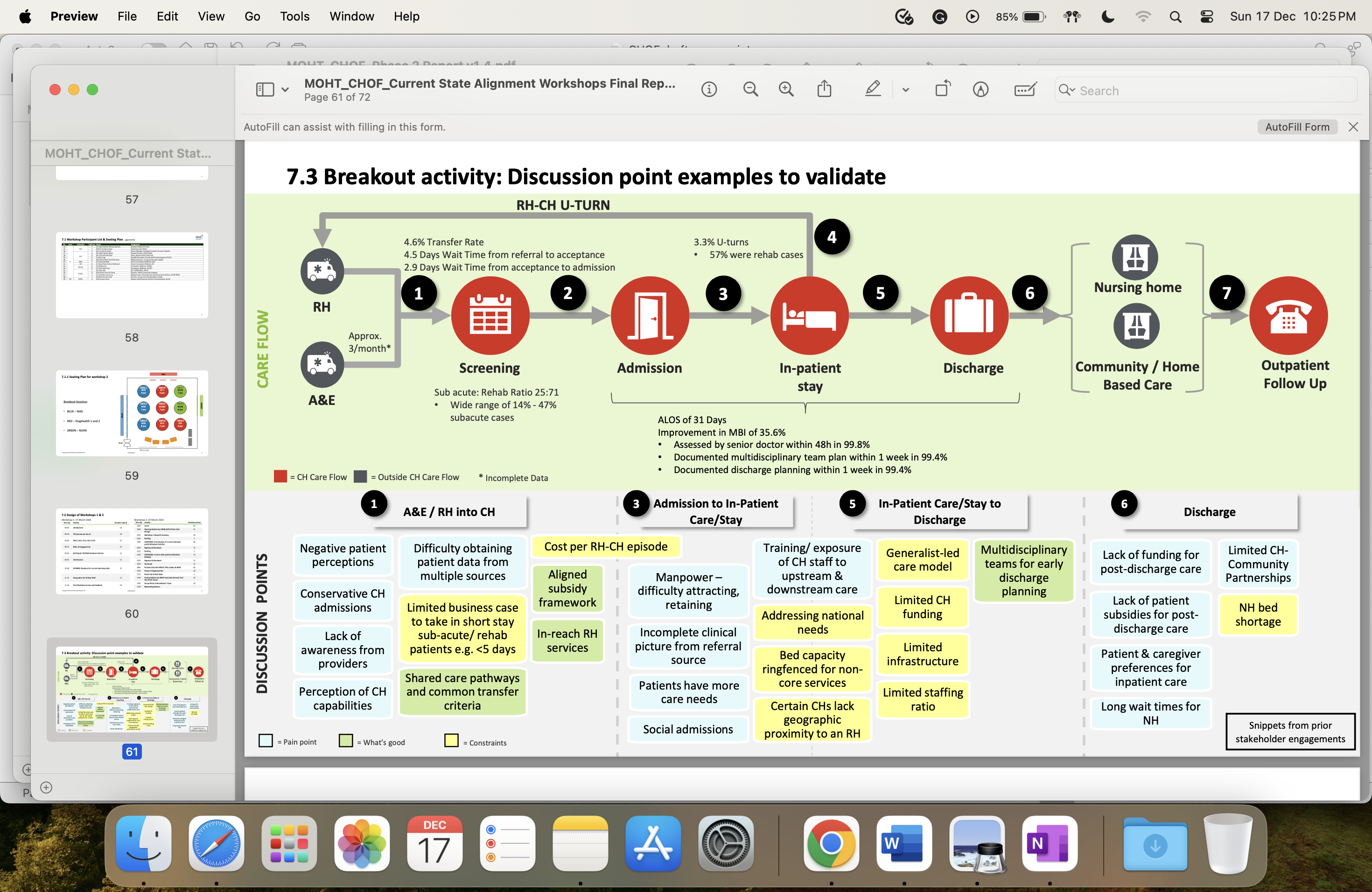
**Figure 2.** Key discussion points from the policy dialogue

Figure 2 summarizes the common pain points, constraints and current strengths experienced by institutions as identified by stakeholders across the four prioritized chokepoints across the patient journey through the community hospital. Similar themes emerged across the chokepoints, including financing, manpower, regulatory, capacity/ infrastructure, and mindsets/ perceptions.


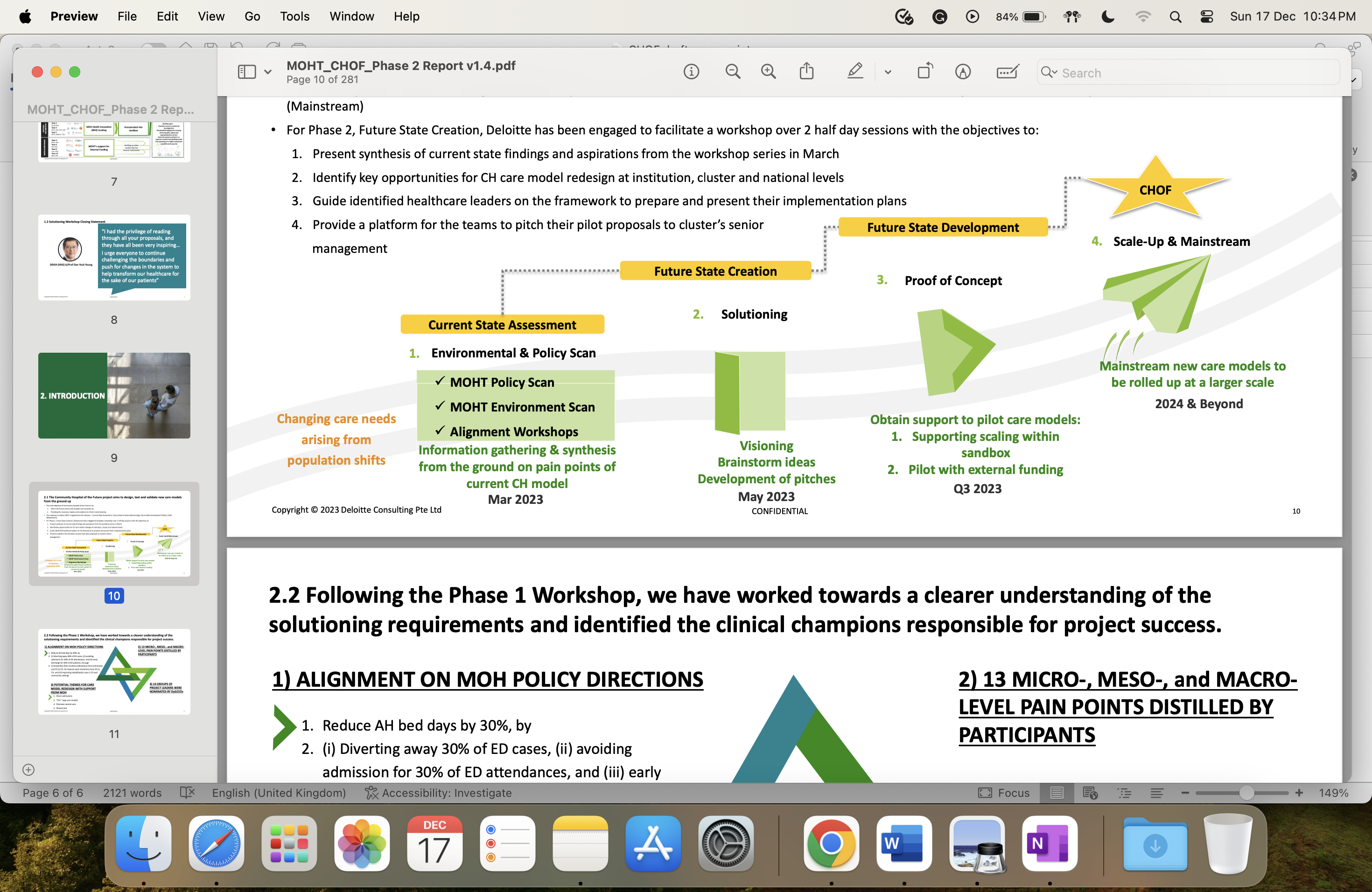
**Figure 3.** Plan of action for policy change
